# Knowledge about Essential Tremor: A Study of Essential Tremor Families

**DOI:** 10.3389/fneur.2018.00027

**Published:** 2018-01-26

**Authors:** Ashley D. Cristal, Karen P. Chen, Nora Cristina Hernandez, Pam Factor-Litvak, Lorraine N. Clark, Ruth Ottman, Elan D. Louis

**Affiliations:** ^1^Division of Movement Disorders, Department of Neurology, Yale School of Medicine, Yale University, New Haven, CT, United States; ^2^Department of Epidemiology, Mailman School of Public Health, Columbia University, New York, NY, United States; ^3^Taub Institute for Research on Alzheimer’s Disease and the Aging Brain, College of Physicians and Surgeons, Columbia University, New York, NY, United States; ^4^Department of Pathology and Cell Biology, Columbia University Medical Center, New York, NY, United States; ^5^G.H. Sergievsky Center, College of Physicians and Surgeons, Columbia University, New York, NY, United States; ^6^Department of Neurology, College of Physicians and Surgeons, Columbia University, New York, NY, United States; ^7^Department of Epidemiology, New York State Psychiatric Institute, New York, NY, United States; ^8^Department of Chronic Disease Epidemiology, Yale School of Public Health, Yale University, New Haven, CT, United States; ^9^Center for Neuroepidemiology and Clinical Neurological Research, Yale School of Medicine, Yale University, New Haven, CT, United States

**Keywords:** essential tremor, clinical, survey, epidemiology, genetics

## Abstract

**Background:**

Essential tremor (ET) is among the most common neurological diseases and it often runs in families. How knowledgeable ET patients and their families are about their disease has been the subject of surprisingly little scholarship.

**Methods:**

To fill this gap in knowledge, we administered a comprehensive 32-item survey (i.e., questions about etiology, pathophysiology, symptoms and signs, natural history, and treatments) to 427 participants, including 76 ET probands, 74 affected relatives (AFRs), 238 unaffected relatives, and 39 spouses of unaffected relatives, all of whom were participating in two ET family studies. We hypothesized that there would be gaps in knowledge about ET and furthermore, that probands and AFRs would be the most knowledgeable, followed by unaffected relatives and then spouses of unaffected relatives, who would be the least knowledgeable.

**Results:**

Overall, ET patients lacked knowledge about their disease. Nearly one-third of probands answered “yes” or “do not know” to the question, “is ET the same or different from the type of tremor that many normal people can get when they become old and frail?” A similar proportion did not know whether children could get ET or they responded “no.” Nearly one-fourth of affecteds (i.e., probands and AFRs) did not know whether or to what degree (e.g., very well, moderately well, not well) the symptoms of ET could be medically controlled, and 38.0% either reported that there was no brain surgery for ET or reported that they did not know. Nearly 17% of affecteds did not endorse genes as a cause for ET, which was surprising given the fact that this was a family study of ET. Probands and AFRs were the most knowledgeable, followed by unaffected relatives. Spouses of unaffected relatives were the least knowledgeable.

**Conclusion:**

We targeted a large group of ET patients and their families, as this group is perhaps most likely to be informed about the disease. ET patients and their AFRs were more knowledgeable about the features of ET than their family members without ET. Overall, however, knowledge of ET was very limited and this lack of knowledge encompassed all aspects of the disease including its underlying causes, the nature of the symptoms and signs, its natural history and its treatment. Further ET awareness education and programs targeting both families of ET patients and the public would help alleviate this gap in knowledge.

## Introduction

Essential tremor (ET) is among the most common movement disorders (1). New research on signs and symptoms, disease progression, risk factors, and treatment has expanded and changed our understanding of ET (2). For example, ET had previously been viewed as a monosymptomatic disorder, but a range of observed non-motor symptoms, such as cognitive impairment and sleep disturbances, has challenged this long-held view (3). For clinicians and patients, keeping up with new ET research findings can be challenging.

Despite being so common, ET is often misdiagnosed by physicians, with studies reporting misdiagnosis in 30–50% of cases (4, 5). A poll of 1,418 ET patients revealed that one-third believed physicians were not educated enough about ET (6). One-hundred and sixteen (9.5%) believed physicians did not satisfactorily discuss and assess issues aside from tremor, and 146 (11.9%) wanted better counseling and management (6). These data suggest that patients desire to learn more than their physicians are able to provide.

Increased knowledge of diseases can empower patients to have more control over their treatment; this can increase patient satisfaction, improve adherence to treatments, and yield better outcomes. In a systematic literature review of patient empowerment in cancer, the authors reported that knowledge is a key element of empowerment, and patients’ understanding of their prognosis and treatment was essential for maintaining a sense of control and engaging in informed decision making (7). Others have reported that patients are more likely to act if they feel the activity has meaning (8). In the context of ET, patients have little control over their tremors and learning more about the disease could help empower them.

How knowledgeable ET patients and the public are regarding the disease has been the subject of only a handful of studies. Two studies focused solely on knowledge of the genetic basis of ET, concluding that most patients were poorly informed (9, 10). A third study examined public knowledge of ET using a more comprehensive questionnaire about the clinical features, causes of, and treatments for ET, but did not question patients themselves (11). No studies have administered a comprehensive questionnaire to ET patients themselves. Furthermore, none has focused exclusively on those with familial ET, which is a group that is more likely to be knowledgeable about the disease.

To fill this gap in knowledge, we administered a comprehensive survey to ET patients and their families. The survey incorporated questions about (1) etiology, genetic underpinnings, and familial risks, (2) pathophysiology, (3) symptoms and signs, (4) distinction from other conditions, (5) natural history and prognosis, (6) comorbidities, and (7) treatments. We surveyed both affected and unaffected family members as well as spouses of unaffected family members, hypothesizing that level of knowledge would follow a specific rank order depending on how distant a family member was from someone affected with ET.

## Materials and Methods

### Subjects and Setting

The 427 participants were enrolled in two ET family studies at Yale University, a family study of the environmental epidemiology of ET (NINDS R01 NS094607, May 2016–present) and the Family Study of Essential Tremor (NINDS R01 NS073872, Phase 2, September 2015–present). The inclusion criteria and enrollment schemes for each study have been described in detail (12). Upon enrollment, participants signed informed consent approved by the Yale University Institutional Review Board. Each study involved a detailed clinical assessment of ET cases and their relatives (both affected and unaffected) using semi-structured questionnaires and a standardized videotaped neurological examination (12). The family study of the environmental epidemiology of ET included only first-degree relatives of a person with ET, whereas the Family Study of ET included both first-degree and second-degree relatives. In the former, unaffected spouses of unaffected relatives were also enrolled. Based on the history and videotaped examination, a senior movement disorders neurologist (Elan D. Louis) assigned ET diagnoses using published diagnostic criteria [moderate or greater amplitude kinetic tremor on ≥3 tests, or head tremor, in the absence of Parkinson’s disease (PD) or dystonia] (12).

### ET Knowledge Questionnaire

As part of their evaluation, all participants were also asked to complete a 32-item questionnaire assessing knowledge about ET. The survey was developed by one of the authors, Elan D. Louis, and included all of the disease-knowledge questions used in a questionnaire that was the centerpiece of a prior study (11) with the addition of 12 new questions relating to disease-knowledge. The questionnaire was piloted on 10 ET patients to assess any ambiguities in wording, and then revised accordingly, thereby arriving at the final version used here. This questionnaire included single choice, multiple-selection, or fill-in-the-blank responses. The questionnaire began with demographic items, including occupation, since some occupations (i.e., health care-related professions) could lead participants to know more about ET. Next, the questionnaire focused on knowledge about (1) etiology, genetics, and familial risks, (2) pathophysiology, (3) symptoms and signs, (4) distinction from other conditions, (5) natural history and prognosis, (6) comorbidities, and (7) treatments.

### Statistical Analyses

All analyses were performed in SPSS statistical software (version 24; SPSS Inc., Chicago, IL, USA). All tests were two-sided and significance was accepted at the 5% level.

Participants were grouped into four categories, with the purpose of determining whether they would differ with respect to knowledge of ET. These were ET probands (*n* = 76), affected first- or second-degree relatives [affected relatives (AFR), *n* = 74], unaffected first- or second-degree relatives (UFR, *n* = 238), and unaffected spouses of unaffected relatives (*n* = 39). The first two groups were assessed separately because of the possibility that probands (i.e., individuals who had been motivated enough to self-refer for participation in the study) might be more knowledgeable about ET.

Age and education were not normally distributed (Kolmogorov–Smirnov test *p* < 0.001 for both). We compared age and education across groups using Kruskall–Wallis tests, and used chi-square tests (or Fisher’s exact test) for additional comparisons of categorical data. To test for trends across the four subject type groups (probands > AFRs > UFRs > spouses), we used logistic regression models, with the response as a binary dependent variable and the four ordinally arranged subject type groups (probands = 1, AFRs = 2, UFRs = 3, and spouses = 4) as the independent variable. Although this yielded odds ratios, 95% confidence intervals, and *p* values, we only report the latter.

## Results

### Demographic Characteristics

There were 427 participants (76 probands, 74 AFRs, 238 UFRs, and 39 spouses of UFRs) (Table 1). Probands were on average approximately 6 years older than other participants. On average, participants completed 16 years of education. Sixty (14.1%) were in a health-related occupation. Among probands, the mean age of tremor onset was 29.2 ± 19.8 years (range = 4–77 years) and the mean tremor duration was 37.5 ± 17.7 years (range = 1–77 years).

**Table 1:** Demographic characteristics of 427 participants.

	Proband	Affected relative	Unaffected relative	Spouse of unaffected relative	All participants	Significance
*N*	76	74	238	39	427	
Age in years	66.2 ± 12.0	61.2 ± 16.9	58.0 ± 13.5	60.1 ± 11.0	60.2 ± 14.0	*p* < 0.001^a^
	Median 66.5	Median 65.5	Median 58.0	Median 61.0	Median 61.0	
Female gender	50 (65.8)	42 (56.8)	159 (66.8)	14 (35.9)	265 (62.1)	*p* = 0.002^b^
Education in years	15.8 ± 2.9	15.6 ± 2.9	16.4 ± 2.9	15.6 ± 5.5	16.0 ± 3.2	*p* = 0.026^a^
	Median 16.0	Median 16.0	Median 16.0	Median 16.0	Median 16.0	
Health-related occupation^c^	9 (11.8)	6 (8.1)	41 (17.2)	4 (10.3)	60 (14.1)	*p* = 0.177^b^

*Number of participants (percentage) or mean ± SD. For age and education, we also report the median*.

*^a^Kruskal–Wallis test*.

*^b^Chi-square test*.

*^c^Physician (*n* = 1), nurse (*n* = 16), home health aide (*n* = 2), student (*n* = 2), medical research (*n* = 6), and other healthcare occupation (*n* = 33)*.

### Etiology, Genetics, and Familial Risks

Three-quarters [325/427 (76.1%)] of the participants endorsed “genes” as a cause for ET. At the same time, 25/150 (16.7%) of affecteds (i.e., probands and AFRs) did not do so, a finding that is somewhat surprising given the fact that this was a family study of ET (Table 2). More than one-third (15/39, 38.5%) of spouses did not endorse “genes” as a cause of ET either. Indeed, there was a clear trend across the four subject type groups (“genes” as a cause of ET in 88.2% of probands, 78.4% of AFRs, 73.9% of UFR, and 61.5% of spouses, Table 2) (test for trend, *p* = 0.001). Only 97/427 (22.7%) participants responded that genes had been identified for ET, which included only 23/76 (30.3%) probands (Table 2). Virtually none of the participants knew that specific genes have been reported as associated with ET in scientific studies (e.g., LINGO1, FUS, ETM) (Table 2).

**Table 2:** Responses of 427 participants to 28 questions about the essential tremor (ET).

	Proband	Affected relative	Unaffected relative	Spouse of unaffected relative	All participants	Significance
*N*	76	74	238	39	427	
**What do you think causes ET? (Indicate one or more)**						
Genes	67 (88.2)	58 (78.4)	176 (73.9)	24 (61.5)	325 (76.1)	***p* = 0.010**^a^
Trauma	9 (11.8)	4 (5.4)	16 (6.7)	0 (0.0)	29 (6.8)	*p* = 0.105^a^
Brain disease	10 (13.2)	7 (9.5)	27 (11.3)	2 (5.1)	46 (10.8)	*p* = 0.394^a^
Abnormal dietary habits	1 (1.3)	4 (5.4)	26 (10.9)	2 (5.1)	33 (7.7)	***p* = 0.033**^a^
Alcohol abuse	6 (7.9)	8 (10.8)	17 (7.1)	1 (2.6)	32 (7.5)	*p* = 0.457^a^
Tobacco abuse	0 (0.0)	1 (1.4)	7 (2.9)	1 (2.6)	9 (2.1)	*p* = 0.443^a^
Environmental toxins	8 (10.5)	8 (10.8)	32 (13.4)	2 (5.1)	50 (11.7)	*p* = 0.477^a^
Medication/drugs	6 (7.9)	6 (8.1)	19 (8.0)	0 (0.0)	31 (7.3)	*p* = 0.339^a^
Systemic metabolic diseases	3 (3.9)	2 (2.7)	15 (6.3)	0 (0.0)	20 (4.7)	*p* = 0.255^a^
Combination of more than one of the above	17 (22.4)	14 (18.9)	68 (28.6)	12 (30.8)	111 (26.0)	*p* = 0.292^a^
Unknown cause	23 (30.3)	24 (32.4)	73 (30.7)	9 (23.1)	129 (30.2)	*p* = 0.768^a^
**Have scientists discovered genes for ET?**						***p* < 0.001**^a^
Yes	23 (30.3)	16 (21.6)	53 (22.3)	5 (12.8)	97 (22.7)	
No	28 (36.8)	12 (16.2)	28 (11.8)	7 (17.9)	75 (17.6)	
Don’t know	25 (32.9)	46 (62.2)	157 (66.0)	27 (69.2)	255 (59.7)	
**Have any of the following genes been linked with ET in scientific studies?**						
LRRK2	0 (0.0)	1 (1.4)	0 (0.0)	0 (0.0)	1 (0.2)	*p* = 0.096^a^
BCDN2	0 (0.0)	0 (0.0)	0 (0.0)	0 (0.0)	0 (0.0)	*p* = 0.086^a^
LINGO1	1 (1.3)	0 (0.0)	10 (4.2)	0 (0.0)	11 (2.6)	***p* = 0.031**^a^
ACTY7	0 (0.0)	0 (0.0)	0 (0.0)	0 (0.0)	0 (0.0)	*p* = 0.086^a^
FUS	0 (0.0)	0 (0.0)	1 (0.4)	0 (0.0)	1 (0.2)	*p* = 0.232^a^
MAPT	0 (0.0)	0 (0.0)	1 (0.4)	0 (0.0)	1 (0.2)	*p* = 0.232^a^
ETM	0 (0.0)	0 (0.0)	2 (0.8)	0 (0.0)	2 (0.5)	*p* = 0.183^a^
DRD3	0 (0.0)	1 (1.4)	2 (0.8)	0 (0.0)	3 (0.7)	*p* = 0.248^a^
HS1-BP3	0 (0.0)	0 (0.0)	2 (0.8)	0 (0.0)	2 (0.5)	*p* = 0.183^a^
**If someone has a parent or sibling with ET, their risk of developing ET is how much higher than someone with ET?**						*p* = 0.151^a^
0× (they don’t have a higher risk)	9 (11.8)	9 (12.2)	16 (6.7)	4 (10.3)	38 (8.9)	
1.5×	2 (2.6)	1 (1.4)	6 (2.5)	3 (7.7)	12 (2.8)	
2×	10 (13.2)	8 (10.8)	19 (8.0)	1 (2.6)	38 (8.9)	
3×	3 (3.9)	2 (2.7)	6 (2.5)	1 (2.6)	12 (2.8)	
4×	2 (2.6)	0 (0.0)	4 (1.7)	0 (0.0)	6 (1.4)	
5×	4 (5.3)	8 (10.8)	15 (6.3)	2 (5.1)	29 (6.8)	
6×	0 (0.0)	1 (1.4)	0 (0.0)	0 (0.0)	1 (0.2)	
8×	0 (0.0)	0 (0.0)	2 (0.8)	0 (0.0)	2 (0.5)	
10×	2 (2.6)	4 (5.4)	8 (3.4)	1 (2.6)	15 (3.5)	
More than 10×	13 (17.1)	6 (8.1)	17 (7.1)	1 (2.6)	37 (8.7)	
Don’t know	31 (40.8)	35 (47.3)	145 (60.9)	26 (66.7)	237 (55.5)	
**Is ET a neurodegenerative disease?**						***p* = 0.026**^a^
Yes	37 (48.7)	27 (36.5)	92 (38.7)	12 (30.8)	168 (39.3)	
No	16 (21.1)	9 (12.2)	24 (10.1)	5 (12.8)	54 (12.6)	
Don’t know	23 (30.3)	38 (51.4)	122 (51.3)	22 (56.4)	205 (48.0)	
**For each of the brain structures, indicate whether scientists believe there is a problem in patients with ET**						
Thalamus	19 (25.0)	6 (8.1)	35 (14.7)	2 (5.1)	62 (14.5)	***p* = 0.044**^a^
Sensory cortex	5 (6.6)	4 (5.4)	17 (7.1)	4 (10.3)	30 (7.0)	*p* = 0.172^a^
Cerebellum	16 (21.1)	7 (9.5)	25 (10.5)	1 (2.6)	49 (11.5)	***p* = 0.003**^a^
Inferior olivary nucleus	2 (2.6)	1 (1.4)	4 (1.7)	0 (0.0)	7 (1.6)	*p* = 0.286^a^
Spinal cord	2 (2.6)	2 (2.7)	8 (3.4)	0 (0.0)	12 (2.8)	*p* = 0.196^a^
Red nucleus	1 (1.3)	1 (1.4)	2 (0.8)	0 (0.0)	4 (0.9)	*p* = 0.206^a^
Supplementary motor area	7 (9.2)	8 (10.8)	23 (9.7)	8 (20.5)	46 (10.8)	*p* = 0.072^a^
**What are the main symptoms of ET?**						
Tremors (unspecified)	36 (47.4)	36 (48.6)	84 (35.3)	11 (28.2)	167 (39.1)	***p* = 0.038**^a^
Shaky arms/hands	37 (48.7)	33 (44.6)	153 (64.3)	23 (59.0)	246 (57.6)	***p* = 0.007**^a^
Shaky head	21 (27.6)	15 (20.3)	77 (32.4)	9 (23.1)	122 (28.6)	*p* = 0.189^a^
Shaky legs/feet	5 (6.6)	4 (5.4)	24 (10.1)	1 (2.6)	34 (8.0)	*p* = 0.276^a^
Shaky voice	18 (23.7)	11 (14.9)	54 (22.7)	6 (15.4)	89 (20.8)	*p* = 0.366^a^
Other	17 (22.4)	12 (16.2)	48 (20.2)	6 (15.4)	83 (19.4)	*p* = 0.706^a^
Do not know	2 (2.6)	3 (4.1)	10 (4.2)	3 (7.7)	18 (4.2)	*p* = 0.649^a^
**What parts of the body can shake when someone has ET?**						
Arms (with or without other)	73 (96.1)	71 (95.9)	233 (97.9)	38 (97.4)	415 (97.2)	*p* = 0.598^a^
Legs (with or without other)	45 (59.2)	35 (47.3)	77 (32.4)	7 (17.9)	164 (38.4)	***p* < 0.001**^a^
Cranial structures (with or without other)	69 (90.8)	65 (87.8)	189 (79.4)	23 (59.0)	346 (81.0)	***p* < 0.001**^a^
Total body	17 (22.4)	12 (16.2)	30 (12.6)	0 (0.0)	59 (13.8)	***p* = 0.030**^a^
**Do patients with ET have problems with balance and walking that are greater than people without ET?**						*p* = 0.665^a^
Yes	48 (63.2)	46 (62.2)	149 (62.6)	23 (59.0)	266 (62.3)	
No	13 (17.1)	7 (9.5)	34 (14.3)	4 (10.3)	58 (13.6)	
Don’t know	15 (19.7)	21 (28.4)	55 (23.1)	12 (30.8)	103 (24.1)	
**Is ET the same or different from the type of tremor that many normal people can get when they become old and frail?**						***p* = 0.020**^a^
The same	11 (14.5)	0 (0.0)	10 (4.2)	4 (10.3)	25 (5.9)	
Similar but not the same	20 (26.3)	19 (25.7)	55 (23.1)	4 (10.3)	98 (23.0)	
Different	34 (44.7)	36 (48.6)	114 (47.9)	20 (51.3)	204 (47.8)	
Do not know	11 (14.5)	19 (25.7)	58 (24.4)	11 (28.2)	99 (23.2)	
**Is ET the same or different from Parkinson’s disease?**						*p* = 0.127^a^
The same	1 (1.3)	1 (1.4)	0 (0.0)	0 (0.0)	2 (0.5)	
Different	74 (97.4)	66 (89.2)	213 (89.5)	32 (82.1)	385 (90.2)	
Do not know	1 (1.3)	7 (9.5)	24 (10.1)	7 (17.9)	39 (9.1)	
**What is the typical age of onset of ET?**						***p* = 0.006**^a^
1–10	4 (5.3)	1 (1.4)	2 (0.8)	0 (0.0)	7 (1.6)	
11–20	3 (3.9)	11 (14.9)	4 (1.7)	2 (5.1)	20 (4.7)	
21–30	7 (9.2)	6 (8.1)	14 (5.9)	2 (5.1)	29 (6.8)	
31–40	3 (3.9)	4 (5.4)	15 (6.3)	1 (2.6)	23 (5.4)	
41–50	8 (10.5)	8 (10.8)	30 (12.6)	3 (7.7)	49 (11.5)	
51–60	8 (10.5)	6 (8.1)	36 (15.1)	7 (17.9)	57 (13.3)	
61–70	4 (5.3)	6 (8.1)	17 (7.1)	5 (12.8)	32 (7.5)	
71–80	1 (1.3)	0 (0.0)	6 (2.5)	0 (0.0)	7 (1.6)	
Any age	25 (32.9)	14 (18.9)	58 (24.4)	5 (12.8)	102 (23.9)	
Do not know	11 (14.5)	17 (23.0)	49 (20.6)	14 (35.9)	91 (21.3)	
**Can children get ET?**						***p* < 0.001**^a^
Yes	58 (76.3)	47 (63.5)	91 (38.2)	10 (25.6)	206 (48.2)	
No	2 (2.6)	2 (2.7)	13 (5.5)	1 (2.6)	18 (4.2)	
Do not know	16 (21.1)	25 (33.8)	134 (56.3)	28 (71.8)	203 (47.5)	
**On average, the tremor of ET progresses (worsens) at a rate of what percent per year?**						*p* = 0.927^a^
It is not progressive	13 (17.1)	23 (31.1)	60 (25.2)	9 (23.1)	105 (24.6)	
<1%	9 (11.8)	7 (9.5)	24 (10.1)	5 (12.8)	45 (10.5)	
1–5%	29 (38.2)	25 (33.8)	89 (37.4)	14 (35.9)	157 (36.8)	
6–10%	11 (14.5)	10 (13.5)	34 (14.3)	6 (15.4)	61 (14.3)	
11–15%	4 (5.3)	1 (1.4)	13 (5.5)	1 (2.6)	19 (4.4)	
16–20%	1 (1.3)	1 (1.4)	6 (2.5)	0 (0.0)	8 (1.9)	
21–25%	1 (1.3)	0 (0.0)	1 (0.4)	0 (0.0)	2 (0.5)	
More than 25%	3 (3.9)	3 (4.1)	5 (2.1)	1 (2.6)	12 (2.8)	
Don’t know	5 (6.6)	4 (5.4)	6 (2.5)	3 (7.7)	18 (4.2)	
**Do patients with ET have an increased mortality compared to people without ET?**						***p* = 0.029**^a^
No	46 (60.5)	36 (48.6)	105 (44.1)	10 (25.6)	197 (46.1)	
Yes, but only slightly	6 (7.9)	4 (5.4)	23 (9.7)	3 (7.7)	36 (8.4)	
Yes, and a lot	1 (1.3)	0 (0.0)	1 (0.4)	1 (2.6)	3 (0.7)	
Don’t know	23 (30.3)	34 (45.9)	109 (45.8)	25 (64.1)	191 (44.7)	
**Do you think people with ET die at a younger age than people who do not have ET?**						*p* = 0.215^a^
Yes	2 (2.6)	3 (4.1)	5 (2.1)	2 (5.1)	12 (2.8)	
No	56 (73.7)	46 (62.2)	157 (66.0)	19 (48.7)	278 (65.1)	
Do not know	18 (23.7)	25 (33.8)	76 (31.9)	18 (46.2)	137 (32.1)	
**What do you think is the average memory/thinking deficit of a patient with ET?**						*p* = 0.086^a^
No problems with memory/thinking	57 (75.0)	50 (67.6)	193 (81.1)	28 (71.8)	328 (76.8)	
Mild problem	19 (25.0)	20 (27.0)	37 (15.5)	9 (23.1)	85 (19.9)	
Moderate problem	0 (0.0)	4 (5.4)	7 (2.9)	1 (2.6)	12 (2.8)	
Severe problem (dementia)	0 (0.0)	0 (0.0)	1 (0.4)	1 (2.6)	2 (0.5)	
**Is ET linked in any way with hearing loss?**						***p* = 0.022**^a^
Yes	0 (0.0)	1 (1.4)	11 (4.6)	1 (2.6)	13 (3.0)	
No	36 (47.4)	34 (45.9)	84 (35.3)	8 (20.5)	162 (37.9)	
Don’t know	40 (52.6)	39 (52.7)	143 (60.1)	30 (76.9)	252 (59.0)	
**Is ET linked in any way with sleeping problems?**						***p* = 0.030**^a^
Yes	11 (14.5)	8 (10.8)	46 (19.3)	4 (10.3)	69 (16.2)	
No	25 (32.9)	18 (24.3)	48 (20.2)	4 (10.3)	95 (22.2)	
Don’t know	40 (52.6)	48 (64.9)	144 (60.5)	31 (79.5)	263 (61.6)	
**Which of the following medical conditions do you think could be related in some way to ET?**						
Epilepsy	8 (10.5)	7 (9.5)	34 (14.3)	5 (12.8)	54 (12.6)	*p* = 0.666^a^
Multiple sclerosis	5 (6.6)	5 (6.8)	14 (5.9)	0 (0.0)	24 (5.6)	*p* = 0.446^a^
Parkinson’s disease	28 (36.8)	23 (31.1)	102 (42.9)	15 (38.5)	168 (39.3)	*p* = 0.313^a^
Alzheimer’s disease	3 (3.9)	5 (6.8)	12 (5.0)	2 (5.1)	22 (5.2)	*p* = 0.891^a^
Neuropathy	9 (11.8)	8 (10.8)	36 (15.1)	5 (12.8)	58 (13.6)	*p* = 0.757^a^
Dystonia	17 (22.4)	9 (12.2)	38 (16.0)	4 (10.3)	68 (15.9)	*p* = 0.253^a^
More than one of the above	0 (0.0)	3 (4.1)	0 (0.0)	0 (0.0)	3 (0.7)	***p* = 0.002**^a^
Uncertain	21 (27.6)	35 (47.3)	108 (45.4)	19 (48.7)	183 (42.9)	***p* = 0.030**^a^
**Is Parkinson’s disease linked in any way with ET?**						***p* < 0.001**^a^
Yes	13 (17.1)	6 (8.1)	41 (17.2)	5 (12.8)	65 (15.2)	
No	42 (55.3)	25 (33.8)	69 (29.0)	10 (25.6)	146 (34.2)	
Don’t know	21 (27.6)	43 (58.1)	128 (53.8)	24 (61.5)	216 (50.6)	
**If a person has ET, what is their risk of developing Parkinson’s disease?**						*p* = 0.370^a^
The same as someone without ET	61 (80.3)	63 (85.1)	199 (83.6)	31 (79.5)	354 (82.9)	
1.5× higher than someone without ET	8 (10.5)	2 (2.7)	11 (4.6)	5 (12.8)	26 (6.1)	
2× higher than someone without ET	2 (2.6)	5 (6.8)	9 (3.8)	1 (2.6)	17 (4.0)	
3× higher than someone without ET	0 (0.0)	1 (1.4)	3 (1.3)	0 (0.0)	4 (0.9)	
4× higher than someone without ET	1 (1.3)	0 (0.0)	3 (1.3)	0 (0.0)	4 (0.9)	
5× higher than someone without ET	0 (0.0)	0 (0.0)	1 (0.4)	1 (2.6)	2 (0.5)	
Less than someone without ET	1 (1.3)	3 (4.1)	8 (3.4)	0 (0.0)	12 (2.8)	
Don’t know	2 (2.6)	0 (0.0)	1 (0.4)	1 (2.6)	4 (0.9)	
**What types of doctors general take care of a patient with ET?**						
General doctor (general practitioner)	27 (35.5)	14 (18.9)	72 (30.3)	6 (15.4)	119 (27.9)	***p* = 0.031**
Internist	9 (11.8)	2 (2.7)	19 (8.0)	3 (7.7)	33 (7.7)	*p* = 0.217
Neurologist	72 (94.7)	65 (87.8)	205 (86.1)	34 (87.2)	376 (88.1)	*p* = 0.252
Neurosurgeon	20 (26.3)	8 (10.8)	33 (13.0)	4 (10.3)	65 (15.2)	***p* = 0.024**
Other	2 (2.6)	1 (1.4)	2 (0.8)	1 (2.6)	6 (1.4)	*p* = 0.625
Not sure	2 (2.6)	9 (12.2)	34 (14.3)	5 (12.8)	50 (11.7)	*p* = 0.054
More than one of the above	33 (43.4)	20 (27.0)	98 (41.2)	10 (25.6)	161 (37.7)	***p* = *0.040***
**Do you think the symptoms of ET can be medically controlled?**						***p* = 0.006**^a^
Yes, very well	4 (5.3)	6 (8.1)	3 (1.3)	0 (0.0)	13 (3.0)	
Yes, with moderate success	35 (46.1)	25 (33.8)	87 (36.6)	13 (33.3)	160 (37.5)	
Yes, but not well	19 (25.0)	10 (13.5)	67 (28.2)	8 (20.5)	104 (24.4)	
No	8 (10.5)	10 (13.5)	22 (9.2)	3 (7.7)	43 (10.1)	
Do not know	10 (13.2)	23 (31.1)	59 (24.8)	15 (38.5)	107 (25.1)	
**Is there some type of brain surgery to treat ET?**						***p* = 0.001**^a^
Yes	55 (72.4)	38 (51.4)	115 (48.3)	14 (35.9)	222 (52.0)	
No	5 (6.6)	9 (12.2)	17 (7.1)	3 (7.7)	34 (8.0)	
Do not know	16 (21.1)	27 (36.5)	106 (44.5)	22 (56.4)	171 (40.0)	
**Could diet and exercise prevent ET or help to control it?**						***p* < 0.001**^a^
Yes	18 (23.7)	16 (21.6)	33 (13.9)	11 (28.2)	78 (18.3)	
No	29 (38.2)	22 (29.7)	47 (19.7)	5 (12.8)	103 (24.1)	
Do not know	29 (38.2)	36 (48.6)	158 (66.4)	23 (59.0)	246 (57.6)	
**Do you think ET is a curable disease?**						***p* = 0.002**^a^
Yes	6 (7.9)	4 (5.4)	14 (5.9)	5 (12.8)	29 (6.8)	
No	51 (67.1)	34 (45.9)	124 (52.1)	10 (25.6)	219 (51.3)	
Do not know	19 (25.0)	36 (48.6)	100 (42.0)	24 (61.5)	179 (41.9)	
**Do you know of a celebrity or historical figure with ET?**						***p* = 0.001**^a^
Yes	33 (43.4)	16 (21.6)	57 (23.9)	5 (12.8)	111 (26.0)	
No	43 (56.6)	58 (78.4)	181 (76.1)	34 (87.2)	316 (74.0)	

*Values are numbers (percentages)*.

*Significant *p* values are in bold (*p* < 0.05)*.

*^a^Chi-square test or Fisher’s exact test*.

Fifty-five percent of participants (237/427) and 31/76 (40.8%) probands did not know the level to which first-degree relatives of affected persons were at increased risk for ET. Indeed, a majority of relatives, including both AFRs and UFRs (180/312 = 57.7%), did not know the level (Table 2). There was a clear trend across the four subject type groups (“did not know” in 40.8% of probands, 47.3% of AFRs, 60.9% of UFRs, and 66.7% of spouses, Table 2) (test for trend, *p* < 0.001).

### Pathophysiology

Nearly one-half of probands (37/76, 48.7%) considered ET to be a neurodegenerative disease (Table 2). Although the thalamus and cerebellum were the two regions most endorsed as the brain region responsible for ET (from a list of seven brain regions), even among probands, only one-fourth or fewer endorsed each of these brain regions (Table 2).

### Symptoms and Signs

Nearly all participants [415/427 (97.2%)] identified the upper limbs as a part of the body that can shake in ET patients while 81% (346/427) identified cranial structures. For cranial structures, there was a clear trend across the four subject type groups (yes to “cranial tremor” in 90.8% of probands, 87.8% of AFRs, 79.4% of UFRs, and 59.0% of spouses, Table 2) (test for trend, *p* < 0.001). Most participants (266/427, 62.3%) and most probands (48/76, 63.2%) thought that ET patients have more problems with balance and walking than people without ET (Table 2).

### Distinction from Other Conditions

Nearly one-third of probands answered “yes” or “do not know” to the question, “is ET the same or different from the type of tremor that many normal people can get when they become old and frail?” (Table 2). Although a full 90.2% of participants (385/427) reported that ET is different from PD, 10/150 (6.7%) participants with ET (i.e., probands and AFRs) thought the two diseases were the same or did not know. Interestingly, as many as 7/39 (17.9%) spouses did not know (Table 2).

### Natural History and Prognosis

Nearly one-half (206/427, 48.2%) of participants acknowledged that children could get ET. Nearly one-third of probands (18/76, 23.6%) either did not know or responded “no,” and three-fourth of spouses (29/39, 74.4%) did the same (Table 2), with a trend across the four groups (test for trend for “do not know,” *p* < 0.001). Most probands responded that ET was progressive, and indicated that the rate of progression was gradual, with the rate most commonly endorsed being 1–5% per year (Table 2). Approximately one-third [23/76 (30.3%)] of probands did not know whether patients with ET have an increased risk of mortality (Table 2) and there was a clear trend across the four groups (30.3% of probands, 45.9% of AFRs, 45.8% of UFRs, and 64.1% of spouses, test for trend *p* = 0.002). The results were similar with the more direct question as to whether ET cases die at a younger age than those without ET (Table 2).

### Comorbidities

Three-quarters [328/427 (76.8%)] of our participants did not think that patients with ET had cognitive difficulty and virtually none [2/427 (0.5%)] thought that patients with ET experience dementia (Table 2). Only thirteen (3.0%) participants thought that ET was linked with hearing loss and only 1/150 (0.7%) affecteds (i.e., probands and AFRs) thought so (Table 2). Similarly, few positively endorsed the presence of sleep problems in ET; more than 60% of participants [263/427 (61.6%)] were uncertain if ET has any links to sleep problems. When asked which medical conditions were related to ET in some way, ET was most commonly linked with PD (36.8% of probands) and with dystonia (22.4% of probands); these values were higher than those for multiple sclerosis, epilepsy, and neuropathy (Table 2). Despite this, the majority of participants (354/427, 82.9%) and probands (61/76, 80.3%) indicated that ET patients were not at increased risk of developing PD.

### Treatments

Few [4/76 (5.3%)] probands thought that the symptoms of ET could be very well controlled medically. Interestingly, 33/150 (22.0%) of affecteds (i.e., probands and AFRs) did not know whether or to what degree (e.g., very well, moderately well, not well) the symptoms of ET could be medically controlled.

Of the affecteds, 57/150 (38.0%) reported that there was no brain surgery for ET or that they did not know. For those who endorsed that there was brain surgery, there was a clear trend across groups (Table 2) (test for trend, *p* < 0.001).

### Other

The majority (316/427, 74.0%) of respondents did not know of a celebrity or historical figure with ET (Table 2) and there was a trend across the four groups (test for trend, *p* < 0.001).

### Additional Analyses

Sixty participants (9 probands, 6 AFRs, 41 UFRs, and 4 spouses) were in a health-related occupation. Because this may have biased the entire sample toward greater levels of knowledge, we repeated our main analyses in the subsample of 367 participants who were not in a health-related occupation. Answers to the questions about the body parts that shake, whether ET is different from PD, whether genes are a cause of ET, whether ET can be medically controlled, and whether there is brain surgery for ET were similar in this group (data not shown).

Affected relatives comprised both first- and second-degree relatives and we tested to see whether level of knowledge was higher in the former. Above we highlighted seven questions for which there was a trend across our four groups. For five of seven of these questions, the AFRs who were first-degree provided responses that exhibited greater knowledge than the AFRs who were second-degree relatives.

We also tested whether severity of tremor in the dominant arm affected knowledge, although did not subject the comparisons to statistical testing due to small sample size. For most comparisons, those with severe tremor had slightly greater knowledge than those with milder tremor. Thus, there were 17 probands who had a tremor rating of 3 on spiral drawing (i.e., severe tremor). Sixteen (94.1%) of these endorsed genes as a cause of ET [compared to 51/59 (86.4%) probands with tremor rating <3]. Among 17 probands with severe tremor, 5 (29.4%) did not know the level to which first-degree relatives of affected persons are at increased risk for ET; among the 59 probands with milder tremor, this value was 26 (44.1%). Fifteen (88.2%) of 17 probands with severe tremor endorsed that there was brain surgery for ET compared with 40 (67.8%) of 59 probands with milder tremor. Three (17.6%) of 17 probands with severe tremor vs. 20 (33.9%) of probands with milder tremor did not know whether patients with ET have an increased risk of mortality. However, for several comparisons, those with more severe tremor did not seem to have more knowledge than those with milder tremor. For example, 15 (88.2%) of 17 probands with severe tremor identified cranial tremor as a part of the body that could be involved vs. 54/59 (91.5%) with milder tremor. Seven (41.2%) of 17 probands with severe tremor vs. 11 (18.4%) of 59 probands with milder tremor either did not know or responded “no” to the question about children getting ET. Seven (41.2%) of 17 probands with severe tremor vs. 26 (44.1%) of 59 with milder tremor knew of a celebrity or historical figure with ET.

## Discussion

Essential tremor is one of the most prevalent neurological diseases in the United States (1). However, it is still a poorly understood disease, especially among the public (11). As such, it does not stand alone. Epilepsy, another highly prevalent neurological disease, is also poorly understood by the public. A mail survey of 4,397 individuals in the United States indicated that the public, in general, had relatively little knowledge of epilepsy (13). Studies of family members of epilepsy patients tend to reveal relatively higher levels of knowledge. A survey of 124 first-degree relatives of epilepsy patients attending a tertiary clinic in Iran indicated that 50% had “good” knowledge about the disease, defined as having a high score on a 25-item questionnaire (14).

Studies similar to ours, of ET patients and/or their families, are few. Fifty ET patients seen by neurologists at Columbia-Presbyterian Medical Center completed a brief survey to assess their knowledge of the genetics of ET, revealing that the majority of these patients were not well informed (15). Similarly, a survey of 111 ET patients and their relatives at a tertiary care center in Singapore concluded that families had little knowledge about ET genetics (10). The authors attributed this result to the possibility that physicians might not be disseminating information because they might “consider ET as ‘benign’ disorder of less urgency or they may be ignorant about the etiology as well” (10). A survey of 250 patients attending various outpatient clinics at Yale New Haven Hospital, including neurology patients and PD patients, concluded that public knowledge about ET was poor (11). No studies have administered a comprehensive questionnaire about the features of ET to ET patients themselves and none has focused exclusively on those with familial ET, which is a group more likely to be knowledgeable about ET.

In the current study, we found that ET patients and their families knew the most basic, observable features of ET (e.g., the presence of hand tremors). However, as the questionnaire progressed through a range of questions about non-motor symptoms in ET, pathophysiology, and prognosis, family members became more uncertain. In other words, knowledge that is being generated in the scientific community is not necessarily filtering down to patients and their families.

We would expect questions regarding genetics and heredity to pique extra interest among family members. However, 16.7% of affecteds did not endorse genes as a cause for ET and this is surprising given the fact that this was a family study of ET whose stated goal in recruitment materials was to try to identify genes for ET. Virtually no participants were familiar with specific genes that have been reported as associated with ET in scientific studies (16–19). Forty percent of probands did not know the degree of increased risk in first-degree relatives of affected persons; published data have indicated that first-degree relatives of ET cases are five times more likely to develop the disease than are members of the general population and 10 times more likely if the proband’s tremor began at an early age (20). While it is possible that patients and families are unaware of current scientific data, the results of gene finding efforts in ET have been mixed (21) and it is possible that ET patients are aware of scientific studies but unable to understand or recall the results.

The results suggest that ET patients lack knowledge even about its clinical features, its natural history, and its distinction from other conditions. Nearly 1 in 3 probands either thought ET was the same as the type of tremor that many normal people can get when they become old and frail or did not know. That is, they did not distinguish the disease from an age-associated trait of normal aging. Nearly 1 in 3 probands either did not know whether children could get ET or responded “no.” Nearly 1 in 4 affecteds (i.e., probands and AFRs) did not know whether ET could be medically controlled and 38.0% of affecteds reported that there was no brain surgery for ET or that they did not know. This may be indicative of poor patient education about the disease and treatment options. Previous studies mentioned this deficiency and the patient’s need for more knowledgeable physicians. For example, a poll of 1,418 ET patients revealed that 31.4% of respondents felt that their doctor was not even moderately well-educated about ET (6).

Probands and AFRs were groups that were most aware of the basic features of ET and were least likely to answer “I don’t know” to questions, suggesting they are more confident in their answers. They may be more motivated to learn more about their disease. When compared with other groups, spouses of unaffected relatives appeared to be the most uninformed and were more likely to answer “I don’t know” on most questions.

Compared to the data presented in Shalaby’s study (11), spouses in this study seem to have more general ET knowledge than the public. This is not unexpected, as they have a family member with ET, although not necessarily a close one. This suggests that some knowledge of the condition is circulating through families and is even reaching spouses of the unaffected relatives. For example, on the question, “Is ET the same or different from PD?” two-third of Shalaby’s participants [69/99 (69.7%)] noted that ET is different from PD while in this study four-fifth of spouses [32/39 (82.1%)] believed the two diseases were different. When asked, “what parts of the body can shake when someone has ET?” 38 (97.4%) spouses identified arms and 23 (59.0%) identified cranial structures. Among Shalaby’s participants, 66 (66.7%) identified arms and 42 (42.4%) identified cranial structures. However, on more advanced topics, such as etiology, the spouses’ knowledge appears similar to the general public’s (11). One caveat, however, with regard to the comparison of the present data to those from Shalaby’s study (11) is that different populations were studied (i.e., the current data were from a family study whereas those of Shalaby were from a survey of 250 patients attending various outpatient clinics at Yale New Haven Hospital). Also, the questionnaires used in the two studies were not identical. These factors could also have contributed to differences between the two studies.

We also tested whether severity of tremor in the dominant arm affected level of knowledge. For most comparisons, those with severe tremor had slightly greater knowledge than those with milder tremor. This is understandable; individuals with more tremor-related dysfunction are more likely as a group to be motivated to learn about their condition.

This study draws attention to a gap in knowledge about ET. One must ask why this gap exists. First, the extent to which this gap in knowledge is derived from the level of knowledge of physicians regarding ET is not clear. How much does the general physician know about ET? How much does the average neurologist know about ET? How much does the typical movement disorders neurologist know about ET? To adequately educate patients and families, physicians must be well aware of recent advances in ET. As a corollary, physicians may be less prone to educate patients regarding issues that are not well established or are controversial, including the diagnosis of ET, its separation from age-related conditions and its association with other degenerative conditions. Second, ET may not be very disabling for some patients, and these patients may be less willing to gain knowledge about the disease. Third, public awareness and social media coverage of ET is less than that of PD. Fourth, the neurological literature is dominated by PD compared to ET.

Are there methods one can use to improve knowledge of ET? The first step is drawing attention to the problem. The second is devising strategies to deal with it. These may include public awareness campaigns (e.g., ET awareness month) as well as awareness campaigns to educate physicians (e.g., distribution of literature, sponsoring of seminars). Although patient advocacy groups are actively engaged in these efforts, lack of significant financial support on the part of the ET community has limited the scope of these efforts. Another problem in ET is the lack of a public figure who is affected and is willing to serve as a public spokesperson for the disease.

The current study should be interpreted within the context of certain limitations. Although the strength of the study was its family design, familial ET cases are not representative of all ET cases, as their knowledge of ET is expected to be higher. Furthermore, collection of data from the public as well as from neurologists would provide additional insights about more widespread knowledge of ET. Also, although the sample size was large, the numbers in certain groups (e.g., spouses) was smaller. Finally, some of the questions and the topics of the questionnaire were difficult and complex (e.g., the names of specific genes) and this could have impacted the ability of patients to demonstrate their knowledge.

Strengths of the study include the overall sample size (*n* = 427). Second, is the uniqueness of the questionnaire and the fact that this is the first study to administer a comprehensive questionnaire to ET patients themselves. Third, it is the first study to focus exclusively on those with familial ET, to gauge the level of knowledge in a group with expected high levels of knowledge. Finally, we surveyed four different types of individuals who *a priori* were expected to exhibit a graded range of knowledge of ET, from probands, who were expected to have the most, to spouses of unaffected relatives, who were expected to have the least.

In conclusion, we targeted a large group of ET patients and their families, as this group is perhaps most likely to be knowledgeable about the disease. ET patients and their AFRs were more knowledgeable about the features of ET compared to their family members who do not have ET. Overall, however, knowledge of ET was very limited and this lack of knowledge encompassed all aspects of the disease, including its underlying causes, the nature of the symptoms and signs, its natural history, and its treatment. Further ET awareness education and programs targeting both families of ET patients and the public would help alleviate this gap in knowledge.

## Ethics Statement

This study was carried out in accordance with the recommendations of the Yale University Institutional Review Board with written informed consent from all subjects. All subjects gave written informed consent in accordance with the Declaration of Helsinki. The protocol was approved by the Yale University Institutional Review Board.

## Author Contributions

AC was involved in the conception and design of this work, the statistical analysis and interpretation of data, the drafting of the manuscript, and gives final approval of this version to be published and agreement to be accountable for all aspects of the work in question. KC was involved in the acquisition of data, the critical revision of the manuscript, and gives final approval of the version to be published and agreement to be accountable for all aspects of the work in question. NH was involved in the acquisition of data, the critical revision of the manuscript, and gives final approval of the version to be published and agreement to be accountable for all aspects of the work in question. PF-L was involved in the conception and design of this work, the critical revision of the manuscript, and gives final approval of the version to be published and agreement to be accountable for all aspects of the work in question. LC was involved in the conception and design of this work, the critical revision of the manuscript, and gives final approval of the version to be published and agreement to be accountable for all aspects of the work in question. RO was involved in the conception and design of this work, the acquisition of data, the critical revision of the manuscript, and gives final approval of the version to be published and agreement to be accountable for all aspects of the work in question. EL was involved in the conception and design of this work, the analysis and interpretation of data, the drafting of the manuscript, and gives final approval of this version to be published and agreement to be accountable for all aspects of the work in question.

## Conflict of Interest Statement

The authors declare that the research was conducted in the absence of any commercial or financial relationships that could be construed as a potential conflict of interest.
